# Quantitative telomerase enzyme activity determination using droplet digital PCR with single cell resolution

**DOI:** 10.1093/nar/gku439

**Published:** 2014-05-26

**Authors:** Andrew T. Ludlow, Jerome D. Robin, Mohammed Sayed, Claudia M. Litterst, Dawne N. Shelton, Jerry W. Shay, Woodring E. Wright

**Affiliations:** 1 Department of Cell Biology, UT Southwestern Medical Center, Dallas, TX 75390-9039, USA; 2 Bio-Rad Laboratories, Digital Biology Center, Pleasanton, CA 94566, USA

## Abstract

The telomere repeat amplification protocol (TRAP) for the human reverse transcriptase, telomerase, is a PCR-based assay developed two decades ago and is still used for routine determination of telomerase activity. The TRAP assay can only reproducibly detect ∼2-fold differences and is only quantitative when compared to internal standards and reference cell lines. The method generally involves laborious radioactive gel electrophoresis and is not conducive to high-throughput analyzes. Recently droplet digital PCR (ddPCR) technologies have become available that allow for absolute quantification of input deoxyribonucleic acid molecules following PCR. We describe the reproducibility and provide several examples of a droplet digital TRAP (ddTRAP) assay for telomerase activity, including quantitation of telomerase activity in single cells, telomerase activity across several common telomerase positive cancer cells lines and in human primary peripheral blood mononuclear cells following mitogen stimulation. Adaptation of the TRAP assay to digital format allows accurate and reproducible quantification of the number of telomerase-extended products (i.e. telomerase activity; 57.8 ± 7.5) in a single HeLa cell. The tools developed in this study allow changes in telomerase enzyme activity to be monitored on a single cell basis and may have utility in designing novel therapeutic approaches that target telomerase.

## INTRODUCTION

The human reverse transcriptase, telomerase, has been studied extensively for the last 20 years for its vital role in aging, stem cells and cancer (1–5). Telomerase is a ribonucleoprotein complex that consists of two core components: a protein component (telomerase reverse transcriptase, hTERT), and a template RNA component (hTERC) that act together to add hexameric 5′-TTAGGG repeats to the ends of linear chromosomes (6). The maintenance of linear chromosome ends (telomeres) is important for cellular survival. The ends of linear chromosomes are similar to deoxyribonucleic acid (DNA) double-strand breaks and thus these ends must be masked otherwise they would form end-to-end fusions and chromosome-bridge-breakage cycles. In addition, when one or a few telomeres reach a critically shortened uncapped length due to the end replication problem (7,8), a DNA damage signal is produced and senescence is induced. Telomerase can delay or prevent telomere length-induced senescence (9–11). The enzymatic activity of telomerase has been widely studied in normal proliferating stem cells and in cancer cells.

The most common assay to measure telomerase activity is the telomere-repeat amplification protocol, or TRAP assay (3). Briefly, a non-5′-TTAGGG-3′_*n*_ substrate for elongation by telomerase (the TS primer derived from a thalassemia breakpoint) is added to a crude lysate (12). Telomerase extends this primer, is then inactivated followed by polymerase chain reaction (PCR) that generates a hexameric ladder of extended products that are visualized on a polyacrylamide gel (13). While the TRAP assay has been widely used, it has several well-recognized shortcomings. First, the TRAP assay is prone to PCR priming artifacts, PCR bias for short fragments and is only able to detect ∼2-fold differences in most laboratories (14–16). In addition, the process generally involves laborious radioactive polyacrylamide gelelectrophoresis (PAGE) and is not conducive to high-throughput analyses. Quantitation of telomerase activity relies on comparison to an internal competitive PCR fragment and is expressed relative to the activity of a reference cell line (that usually varies between different laboratories). A more precise and reproducible quantitative assay is needed to assess the efficacy of interventions that inhibit or activate telomerase in the range of 20–30%. This degree of precision is important but with current techniques is not feasible. Recently, a novel PCR technology, droplet digital PCR (ddPCR), has been developed that allows for highly reproducible absolute quantification (molecule counting) of input DNA molecules.

ddPCR partitions a sample across a population of uniformly sized emulsified droplets (17,18). The droplets are subjected to endpoint PCR thermocycling so that plateau is reached. Thus the rate of amplification is unimportant, only whether or not amplification has occurred. The total number of droplets and the ratio of template positive (fluorescent) to template negative (no fluorescence) is then determined (hence the description of the results as digital; a droplet is either + or −) in a flow cytometer-like fashion (17,18). This number is then corrected using Poisson statistics, incorporating the probability of having multiple copies of target molecules in one droplet. One can achieve improved metrics (sensitivity, dynamic range, precision and reproducibility) with the large number of digital reactions that are performed (∼20 000 nl-sized droplets). This technology produces precision detection of 1–100 000 molecules per reaction, and as many reactions as needed can be processed per sample (17). These properties make ddPCR uniquely suited for adaptation of the TRAP assay to this molecule-counting platform. The recent development of the QX200 ddPCR™ system compatible with the intercalating dye EvaGreen^®^ made it possible to adapt the TRAP assay for ddPCR. Since further processing of the sample is not needed after PCR (i.e. PAGE, imaging and gel quantification), ddPCR makes determination of telomerase activity at a much higher throughput compared to gel-based methods. Here we describe the reproducibility, linearity and utility of quantitative telomerase enzyme activity detection using a ddPCR approach we term the ddTRAP assay (droplet digital TRAP assay).

## MATERIALS AND METHODS

### Tissue culture

*Telomerase positive cells*: HeLa (cervical carcinoma), H1299 (lung adenocarcinoma), A549 (lung carcinoma), HT116 (colorectal carcinoma), NIH 3T3 (spontaneous immortal mouse fibroblasts) and SW39 (IMR90 T-antigen immortalized fibroblasts). *Telomerase negative ALT cell lines (Alternative Lengthening of Telomeres)*: U2OS (osteosarcoma), SW13 (IMR90 T-antigen immortalized fibroblasts), GM847 (SV40 infected human skin fibroblasts), MDA087 (human mammary stromal cells), SAOS (osteosarcoma), VA13 (SV40 infected human lung fibroblasts) and SKLU-1 (lung adenocarcinoma). *Telomerase negative normal cell strains*: BJ (foreskin fibroblasts), IMR90 (fetal lung fibroblasts), BJ E6E7 (transformed pre-crisis thus not immortal fibroblasts). All cell lines were cultured in ambient oxygen, 5% CO_2_ and maintained in a 4:1 ratio of Dulbecco's modified Eagle's medium to Medium 199 containing 10% calf serum (HyClone, Logan, UT, USA).

### Primary human lymphocyte isolation, culture and stimulation

Antecubital vein blood was drawn into a 10 ml ethylene diaminetetraacetic acid (EDTA) Vaccutainer, as previously described (19). One part blood was mixed with three parts 1× Phosphate buffered saline (PBS) and layered over Ficoll (Ficoll-Paque, Amersham-Pharmacia) and centrifuged (300 × g) for 30 min at room temperature. Mononuclear cells were collected, washed in 1× PBS and pelleted at 300 × g for 10 min at room temperature. Cells were re-suspended in 10 ml of 20% fetal bovine serum (FBS) in 1640 RPMI Glutamax media (Life Technologies, Invitrogen, Carlsbad, CA, USA) supplemented with antibiotics (penicillin and streptomycin) and counted (20). One hundred thousand cells were plated into individual T-25 Primaria tissue culture flasks (BD Biosciences, San Jose, CA, USA) in duplicate for each time-point and condition. One half was stimulated with PHA (1 μg/ml, Sigma-Aldrich) for the indicated period of time prior to isolation and lysis of cells (details listed below and in figure legends).

### Experimental strategy and assay workflow

**Figure 1.**
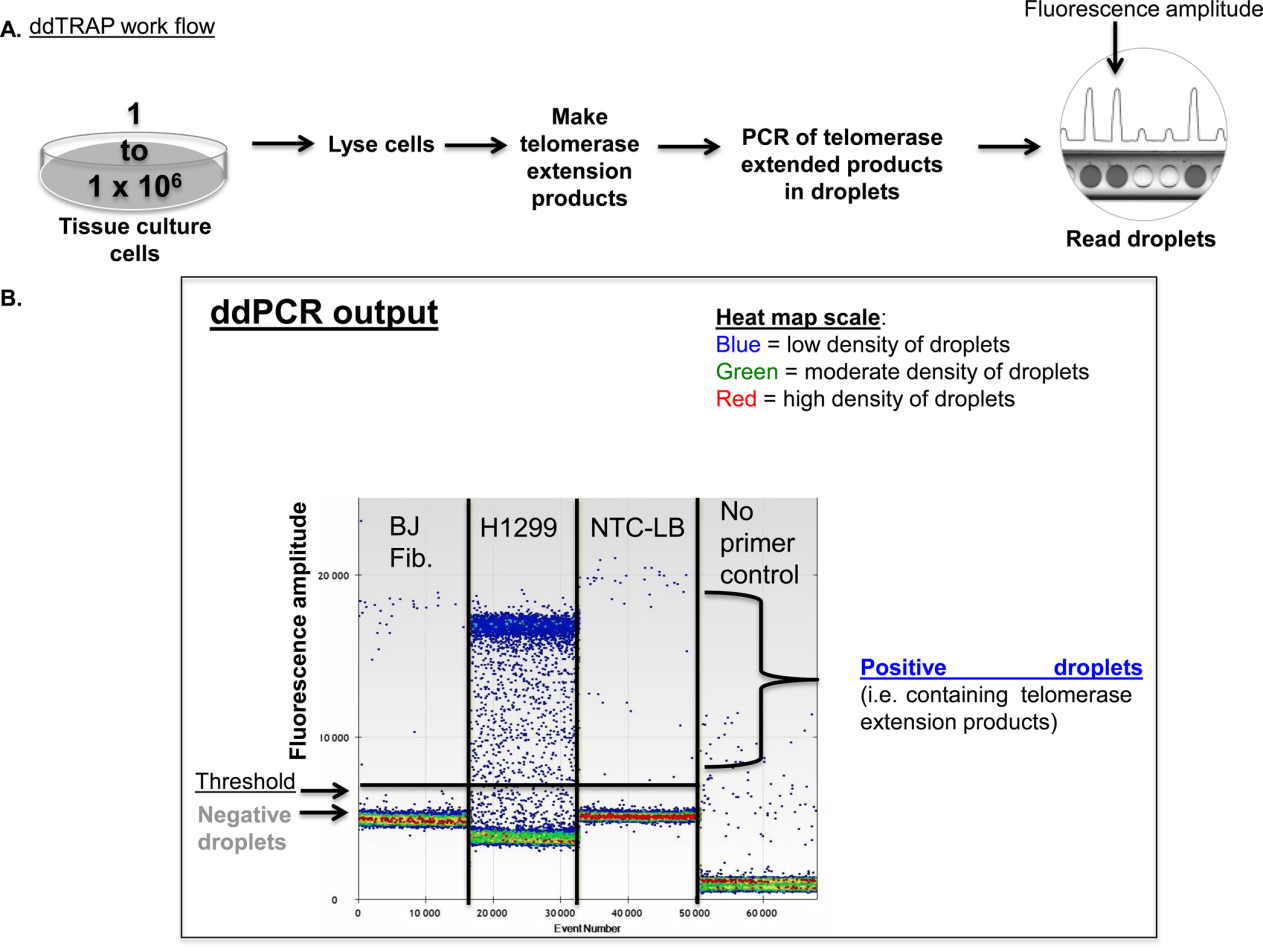
Workflow and optimization of droplet digital TRAP. (**A**). The ddTRAP workflow. Cells are lysed at a concentration of 1250 cells per μl, telomerase extension products generated at a concentration of 25 cells/μl, then telomerase is heat inactivated and extension products dispersed into droplets. PCR thermocycling is done for 40 cycles and droplets analyzed for the presence or absence of fluorescence by the droplet reader. (**B**). ddTRAP output showing BJ fibroblasts (input of 100 cell equivalents, telomerase negative), H1299 cells (input of 100 cell equivalents, telomerase positive), a lysis buffer only control and a control with no primers and input of 100 cell equivalents of H1299 lysate to test for specificity of amplification. Only very low background signals are seen in these controls. Each well or sample of the ddPCR analyzes about 17 000 droplets. Event number at the bottom of the output represents the number of droplets counted in the wells overtime. Each dot on the ddPCR output represents a unique droplet that is either positive or negative for fluorescent signal. Fluorescence amplitude is a measure of the fluorescence detected for each droplet in the assay. Fluorescence amplitude is used to separate the positive and negative droplets. Since the droplets are detected with EvaGreen^®^ double stranded DNA binding dye there will be inherent background fluorescence of DNA molecules not amplified during PCR. The heat map scale represents the density of droplets at given fluorescent amplitudes. NTC-LB = no-template control-lysis buffer.

Cells were cultured to the desired density, counted (50 000 to 1 × 10^6^ cells), and pelleted. The cell pellets were either placed on ice and lysed immediately or flash frozen in liquid nitrogen and stored at −80°C for later use. Cells were lysed in 40 μl of NP-40 buffer (10 mM Tris-HCl, pH 8.0, 1 mM MgCl_2_, 1 mM EDTA, 1% (vol/vol) NP-40, 0.25 mM sodium deoxycholate, 10% (vol/vol) glycerol, 150 mM NaCl, 5 mM β-mercaptoethanol; 0.1 mM AEBSF (4-(2-aminoethyl)benzenesulfonyl fluoride hydrochloride) for a minimum of 30 min on ice. The lysate cell number equivalent was calculated by dividing the number of cells lysed by the volume of lysis buffer used (e.g. 50 000 cells/40 μl = 1250 cells/μl). One microliter of this lysate was added to a 50 μl extension reaction containing 1× TRAP reaction buffer (10× concentration: 200 mM Tris-HCl, pH 8.3, 15 mM MgCl_2_), 0.4 mg/ml BSA, TS telomerase extension substrate (HPLC purified, 200 nM; 5′-AATCCGTCGAGCAGAGTT), dNTPs (2.5 mM each) and incubated for 40 min (or as listed in the figure legends) at 25°C followed by telomerase inactivation at 95°C for 5 min, then held at 4°C. The ddPCR reaction was assembled containing 1× EvaGreen ddPCR Supermix v2.0 (Bio-Rad, Hercules, CA, USA), 50 nM TS primer, 50 nM ACX primer, 50 cell equivalents or less of extension product and dH_2_O to 20 μl per sample. Droplets were produced in the droplet generator according to the manufacturer's instructions (Bio-Rad, Hercules, CA, USA), the emulsions transferred to a 96-well PCR plate (twin-tec 96-well plate, Eppendorf, Fisher) and sealed with foil (Thermo Scientific, AB0757). More specifically, the 20 μl PCR reaction was pipetted into the center well of a universal droplet generator cartridge^®^ (QX100/200 droplet generation cartridges, Bio-Rad). In addition to the PCR reaction 70 μl of droplet generation oil^®^ (EvaGreen droplet generation oil, Bio-Rad) was added to the left well of the cartridge prior to being placed into the droplet generator (QX100 drop generator, Bio-Rad). Following droplet generation, 40 μl of emulsion was transferred from the right well of the cartridge and placed into a 96-well PCR plate. PCR was performed (T100 thermocycler, Bio-Rad, Hercules, CA, USA) with a ramp rate of 2.5°C/s between all steps. Activation of Taq polymerase (95°C for 5 min) was followed by 40 cycles of 95°C for 30 s, 54°C for 30 s, 72°C for 30 s, then held at 12°C. Following PCR, the fluorescence was read on the droplet reader (QX200, Bio-Rad, Hercules CA) using the 6-Fam channel (channel 1 on the software). On average 17 000 droplets were analyzed per 20 μl PCR. The threshold between positive and negative droplets was determined by including controls such as a no-template lysis buffer control (NTC-LB) (Figure 1A and B) and a control where no primers were present but lysate was added to the ddPCR (a test for specific amplification). Once the threshold (i.e. separation of positive droplets and negative droplets) was determined the output was given in molecules of extension products per microliter of ddPCR reaction. We define telomerase activity as the number of extended TS molecules counted by ddPCR and expressed in molecules per microliter. The measured telomerase activity was then converted to total product generated (i.e. number of extension products per microliter from the ddPCR readout multiplied by 20 μl). The telomerase activity was normalized to a per-cell equivalent basis by dividing total product generated by the number of cell equivalents input into the assay. For example, if 50 000 cells were lysed in 40 μl (1250 cells/μl), and 1 μl of lysate was added to a 50 μl extension reaction (25 cells/μl) and 2 μl of the extension reaction was added to the ddPCR (50 cell equivalents) then the total telomerase products generated would be divided by 50. The background signal was determined by including a NTC-LB. The NTC-LB reaction was included in the extension and ddPCR steps to control for a very minor background signal generated during sample preparation. The average background signal across experiments was 1.5 ± 0.81 molecules/μl (mean ± standard deviation; Poisson corrected average upper and low 95% confidence intervals 2.49 and 1.21 molecules/μl). For each plate the NTC-LB concentration (molecules/μl) was subtracted from each unknown sample's concentration prior to further analysis.

### Linearity and reproducibility experiments

We generated telomerase extension products across a range of cell equivalents to determine the reproducibility and linearity of ddTRAP. One million HeLa cells were lysed in 40 μl of lysis buffer resulting in a concentration of 25 000 cell equivalents/μl. Samples were then diluted in lysis buffer 1:10 (2500 cell equivalents/μl) and then diluted 1:2 in serial dilutions prior to extension in a 50 μl reaction (thus final concentrations of 50, 25, 12.5 6.25 and 3.125 cell equivalents/μl of extension reaction). Two microliters of each extension reaction was added to ddPCR reactions. The final cell equivalents of HeLa in the ddPCR reactions were 100, 50, 25, 12.5 and 6.25. To test if ddTRAP could detect telomerase activity down to a single cell equivalent, HeLa cell extracts from 50 000 cells were diluted to produce extension reactions containing 12.5, 6.25, 3.13, 1.78 and 0.78 cell equivalents/μl.

### Gel-based TRAP methods

Telomerase enzyme activity was determined using the identical lysis condition and extension conditions as described above and used previously from our laboratory (13). For gel-based determinations, 5 μl of extension reaction was mixed with a PCR reaction containing the following HPLC purified primers (TS, ACX, TSNT and NT) as previously described (21). The reaction products were then resolved by 10% PAGE and visualized. The images were then processed and quantified using either ImageJ or Syngene gel quantification software (22).

### Single cell methods

To perform single cell experiments we first determined a mixture of lysis buffer and extension reaction mixture that gave comparable results to our typical methods. The NP40 lysis buffer described above was treated as 2.5× and mixed with the components for the extension reaction (10× TRAP buffer, TS substrate, dNTPs, BSA and dH_2_O) in a final volume of 50 μl. To determine if this reaction was linear, we performed a series of reactions containing different numbers of cells (100 000, 50 000, 25 000, 4000, 2000 and 1000 cells). The lysis-extension mixture with cells was placed on ice for 30 min prior to being placed into a thermocycler for telomerase extension (40 min at 25ºC), followed by enzyme inactivation (95ºC for 5 min) and then held at 4ºC until ddPCR. These products (2 μl) were then added to ddPCR and the number of extended TS substrates quantified as described above. Next, we plated 2 μl of two dilutions of cells (3000 cells/μl and 300 cells/μl) in 6-well plates and allowed the cells to adhere overnight. This was done to achieve cells distributed in the wells at low enough density to allow single cells to be visualized with a light microscope and picked with a pipette containing 2 μl of lysis-extension mixture (described above). We picked single cells (*n* = 78) and various other numbers of cells from two cells to eight cells. The 2 μl lysis-extension reaction was pipetted into a PCR tube, placed on ice immediately following extraction from the 6-well tissue culture plate, and allowed to incubate for at least 30 min. Following lysis on ice, the reaction was placed in a thermocycler for the telomerase extension reaction for 40 min. Following extension, 18 μl of ddPCR mixture (10 μl 2× EvaGreen ddPCR Supermix^®^, 0.1 μl of 10 μM TS primer, 0.1 μl of 10 μM ACX primer and 7.8 μl of dH_2_O) was added to the 2 μl lysis-extension reaction, droplets were produced, PCR performed and fluorescence of the droplets read as described above. The ddPCR in copies per microliter was corrected for background (subtraction of the copies per microliter value of the no-template lysis buffer). The corrected copies per microliter values were then multiplied by 20 to give the total telomerase products values, if a single cell was assayed we assumed this to equal the amount of telomerase activity per-cell, with activity being measured by the number of extended TS molecules counted during ddPCR analysis.

### Imetelstat (telomerase inhibitor) IC50 dose experiment

HeLa cells (50 000 cells) were plated in three independent 6-well tissue culture plates and allowed to attach overnight. Cells were then treated with Imetelstat for 72 h. Cells were then counted, pelleted and lysed in 40 μl and diluted in triplicate prior to extension and telomerase enzyme activity determination by gel based TRAP and ddTRAP.

## RESULTS

### Optimization of the ddTRAP reaction

**Figure 2.**
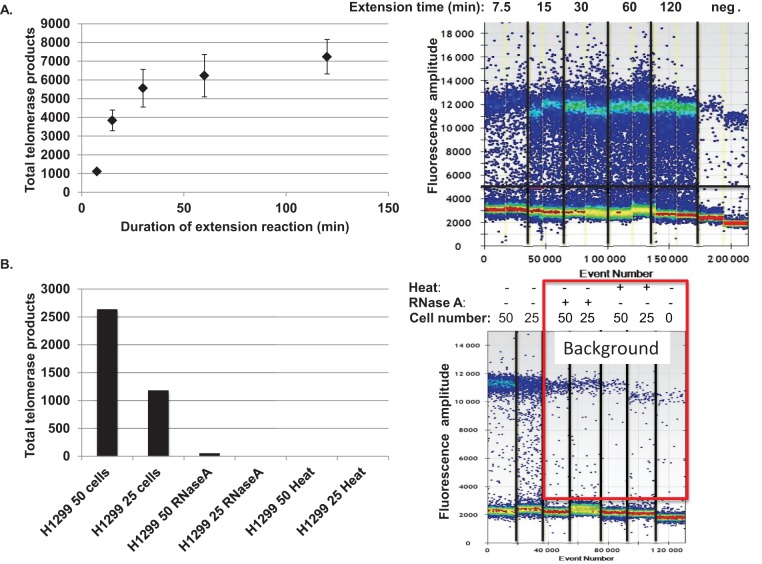
Optimization of ddTRAP. (**A**) ddTRAP fluorescent droplet outputs are shown on the right, and quantified outputs graphed on the left. Lysates were incubated with TS substrate for various amounts of time prior to heat inactivation, using 100 cell equivalents from H1299 cells. Data are presented as means of background corrected total telomerase products generated ± standard error of the mean. Background corrected total telomerase products generated was calculated by first multiplying the concentration of molecules counted (molecules per microliter) by 20 (for the 20 μl PCR) and then subtracting the mean of the NTC-LB control from each sample. (**B**) Heat and RNase A inactivation of telomerase resulted in virtually no ddTRAP signal. This permitted a small background correction to be included, and established the specific measurement of telomerase activity from lysates. Background signal is highlighted by the red box.

We first examined telomerase activity as a function of extension reaction duration. Figure 1A and B show the overall workflow and how the controls (NTC-LB and no primer controls) help determine the correct threshold between positive and negative droplets. Figure 1B highlights the robust difference the ddTRAP assay detects between telomerase negative (BJ fibroblasts) and telomerase positive cells (H1299 lung cancer cells). The detection of telomerase extension products increased exponentially from 7.5 to 30 min and then slowed but did not plateau for up to 2 h (Figure 2A). For typical applications a 40 min extension reaction that detects ∼75% of the 2 h activity is sufficient. Several negative control reactions (Figure 2B, heat and RNase A negative controls, lysis buffer only for contamination and no primer control to test for specific amplification) showed that the vast majority of the signal detected in the droplets was specific to telomerase activity. These controls provided a small value for correcting for this background (see explanation of background correction in ‘Materials and Methods’ section). Both RNase A and heat abolished detectable levels of telomerase activity by ddTRAP as expected. We extracted the DNA from the droplets and analyzed them on a polyacrylamide gel: the expected 6-bp TRAP ladder pattern was observed, indicating that ddTRAP does assay telomerase extension products (Supplementary Figure S1).

### ddTRAP is linear and reproducible

**Figure 3.**
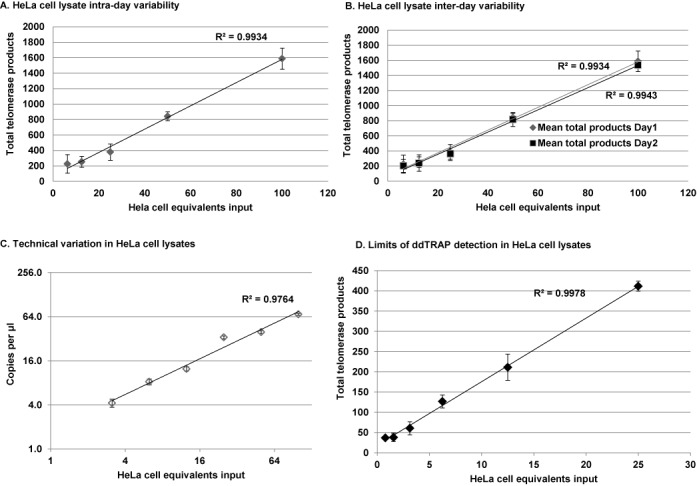
Reproducibility and linearity of ddTRAP. (**A**) Intra-day variability was assessed by making three extension reaction dilution series and assaying telomerase activity on one plate for each cell input (three replicates per-cell equivalent input). A clone of the HeLa cell line specific to our lab observed to have intermediate telomerase activity (was used in this experiment see Figures 4 and 6 for HeLa cell population telomerase activity). Data are plotted as total telomerase product (copies of target molecules per microliter multiplied by 20 = copies per μl) by cell equivalents. Error bars are standard deviation of the replicates on each plate. (**B**) Inter-day variability was assessed by running two assays on two separate days from the same three extension reactions described in (A). Data are plotted as total telomerase product (copies per microliter multiplied by 20) by cell equivalents. Error bars are standard deviation of the replicates on each plate. (**C**) Technical variation was assayed by diluting the 100 cell equivalent sample 1:2 in a series of six samples. Telomerase activity was measured on two separate plates on the same day. Technical variation is plotted in copies per microliter (output from ddPCR software) by cell equivalents input on a log scale. Error bars are Poisson corrected 95% confidence intervals across the two plates. (**D**) HeLa cell dilution series from 50 to 1 cell equivalents produced a linear relationship (*R*^2^ = 0.99) between input and detection of telomerase enzyme activity as indicated by total product generated in the ddPCR. Error bars are the standard deviation of the replicates. Although the extract was not made from a single cell, ddTRAP was able to reproducibly detect telomerase activity above background at the dilution equivalent to one cell input.

**Figure 4.**
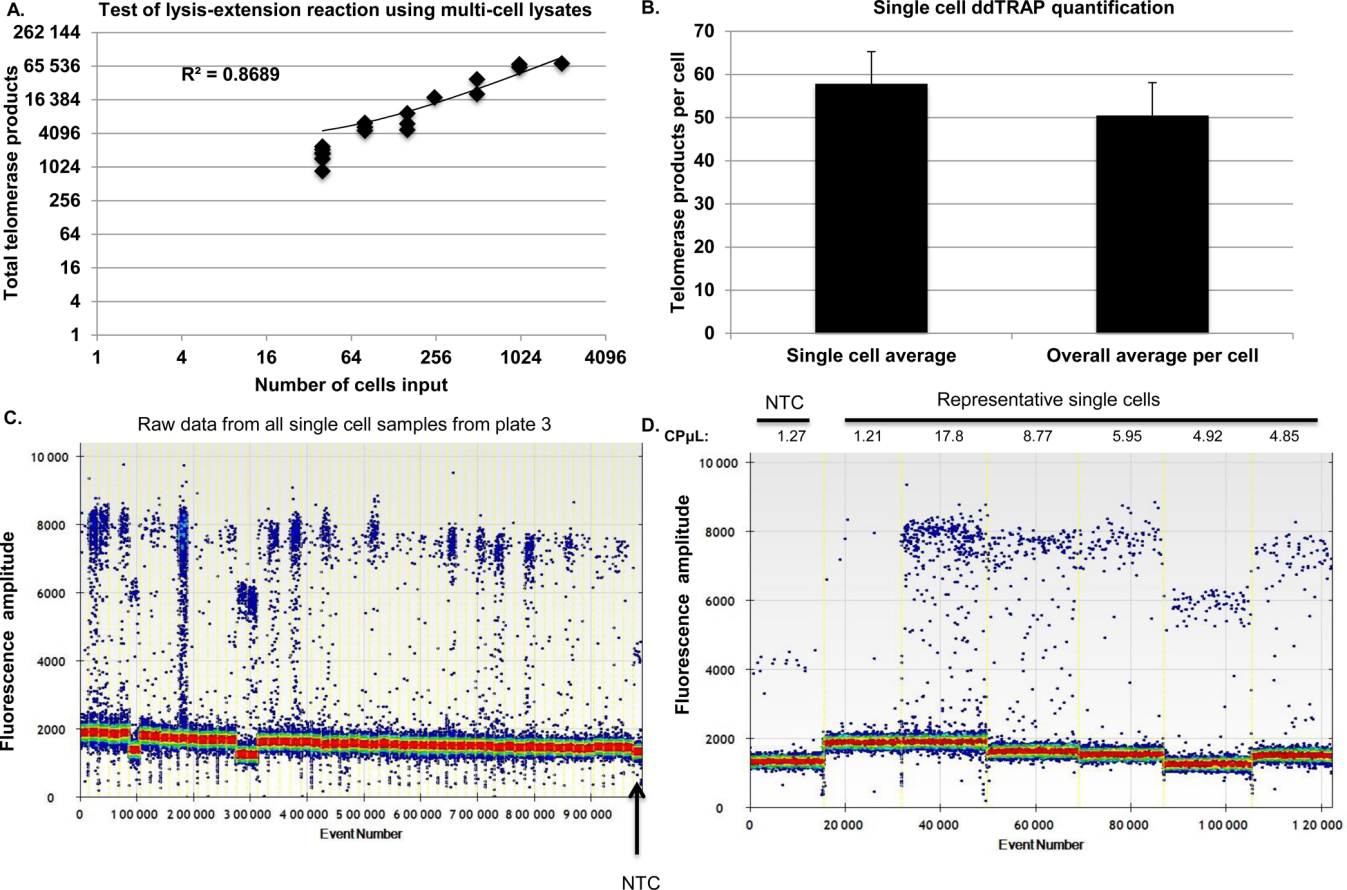
Telomerase activity in single cells. Telomerase activity (i.e. the number of extended TS molecules counted following ddPCR) in single cells was determined using a lysis and extension reaction. (**A**) The lysis-extension buffer is linear and produced similar results to the two-step lysis-extension procedure previously used. The slightly higher values are likely due to more efficient lysis using this new buffer system. Note that the scale of the graph is log_(4)_. (**B**) Telomerase activity from single HeLa cells. 2 μl of two dilutions of cells (3000 cells/μl and 300 cells/μl) were plated overnight in 6-well plates to allow single cells to be visualized with a light microscope, then dissolved in 2 μl of lysis-extension mixture. We picked both single cells (*n* = 78) and groups of 2–8 cells. The 2 μl lysis-extension reaction was incubated on ice for at least 30 min, then placed in a thermocycler for the telomerase extension reaction at 25ºC for 40 min. Following extension, 18 μl of ddPCR mixture (10 μl 2× EvaGreen ddPCR Supermix^®^, 0.1 μl of 10 μM TS primer, 0.1 μl of 10 μM ACX primer and 7.8 μl of dH_2_O) was added to the 2 μl lysis-extension reaction, droplets were produced, PCR performed and fluorescence of the droplets read. The ddPCR in copies per microliter was corrected for background (subtraction of the copies per microliter value of the no-template lysis buffer control). The corrected copies per microliter values were then multiplied by 20 to give the total telomerase products values. An average of 57.8 telomerase-extended molecules were observed per-cell. Telomerase activity assayed was similar in single cell lysates compared to telomerase activity detected in lysates generated from greater numbers of cells using the lysis-extension buffer and normalized to a per-cell equivalent average (range from 2 to 8 cells). (**C**) Raw ddPCR output of all single cell lanes from the third 96-well plate that was run using the lysis extension system. (**D**) Representative lanes of ddPCR output showing the NTC-LB control, a negative sample and several samples of various telomerase activities. CPμl = copies of target per microliter, output from the droplet reader software.

To determine intra- and inter-day reproducibility of ddTRAP we tested dilutions of different numbers of cell equivalents added into the extension reaction. For HeLa and H1299 cells intra- and inter-plate technical replicates produced coefficients of variation (CV) that were dependent upon the cell equivalents added to the ddPCR reaction. The CVs varied between 5.1 and 47.4% (HeLa Figure 3A and B, Tables 1 and 2; H1299 Supplementary Figure S2A and B). For HeLa cells, adding 50 cell equivalents to the ddPCR reaction resulted in the most consistent intra- and inter-plate CVs of 6.9 and 9.1%, respectively. For H1299 cells, adding 50 cell equivalents to the ddPCR reaction also produced the most consistent CVs (Supplementary Figure S2). We chose 50 cell equivalents in the majority of the remainder of our analyses. These CV values (inputs ranging from 500 to 1 cells for HeLa, Figure 3 and Supplementary Figure S3 and 50 to 3 cells for H1299 Supplementary Figure S2), shows the large dynamic range of cell inputs for the ddTRAP assay. We also assessed the technical variation of ddTRAP and observed very small Poisson corrected confidence intervals, indicating that technical variation introduced by ddPCR is minimal (Figure 3C and Supplementary Figure S2C). We were able to detect activity in a single HeLa cell equivalent (Figure 3D). Although the extract was not made from a single cell, this indicates the potential for single cell detection of telomerase activity utilizing ddTRAP (as shown in Figure 4). These three dilution series had *R*^2^ values of 0.98–0.99, indicating the ddTRAP assay is linear and able to detect less than 2-fold changes in telomerase activity (Figure 3).

**Table 1.** Intra-day coefficients of variation in HeLa cells

HeLa cell equivalents	Average concentration (total telomerase products)	Standard deviation	%CV
100	1587.3	136.6	8.61
50	842.0	57.7	6.85
25	378.7	106.1	28.02
12.5	252.0	71.1	28.21
6.25	225.1	118.7	52.72

Data are presented from three dilution series assayed on the same plate. CV = coefficient of variation.

**Table 2.** Inter-day coefficients of variation in HeLa cells

HeLa cell equivalents	Average concentration (total telomerase products)	Standard deviation	%CV
100	1562.0	81.2	5.1
50	829.7	75.0	9.1
25	371.0	89.6	24.1
12.5	245.5	90.5	37.1
6.25	213.8	101.9	47.4

Data are presented from three dilution series assayed on two different plates. CV = coefficient of variation.

### Telomerase enzyme activity in single cells

To determine telomerase activity in single cells, we devised modified lysis-extension conditions. Figure 4A shows that the lysis-extension conditions are linear (*R*^2^ = 0.86). We picked single HeLa cells (*n* = 78) using a lysis-extension reaction and determined that 57 of the 78 singles cells (73%) produced detectable ddTRAP signal above background (Figure 4B), indicating but certainly not proving that about 30% of the cells in a given HeLa cell lysate may be telomerase negative. The average number of telomerase-extended products detected in a single cells was 57.8 ± 7.5. The number of telomerase-extended products is slightly greater than that observed in the standard two-step ddTRAP reaction likely due to more efficient lysis, further tests are needed to evaluate different lysis conditions in ddTRAP.

**Figure 5.**
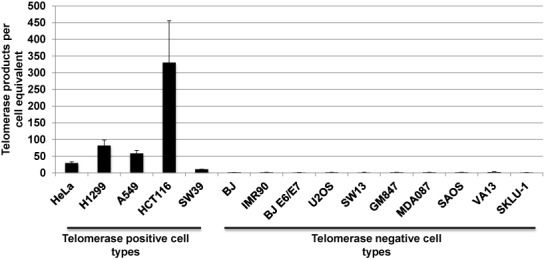
Telomerase activity across different cell types. Cancer cell lines known to be telomerase positive gave ddTRAP signals while cell lines known to be telomerase negative gave no signal following background correction. 50 cell equivalents from each cell line were analyzed with ddTRAP. Telomerase activity is defined as the number of extended TS molecules counted following ddPCR and then multiplied by 20 and divided by the number of cell equivalents added into the PCR to produce normalized activity values.

We assayed telomerase activity in a panel of telomerase positive and negative cancer cell lines and normalized the activity to the number of cell equivalents added to the assay. As expected telomerase positive cancer cells (i.e. HeLa) produced a robust signal in the ddTRAP reaction compared to the absence of signal in telomerase negative cell lines such as BJ and IMR90 fibroblasts, human papillominavirus (E6/E7) transformed BJ fibroblasts and alternative lengthening of telomeres (ALT) cancer cells (Figure 5; (23); qualitative gel-images of most cell lines used are shown in Supplementary Figure S4).

### Comparison of ddTRAP to gel-TRAP

**Figure 6.**
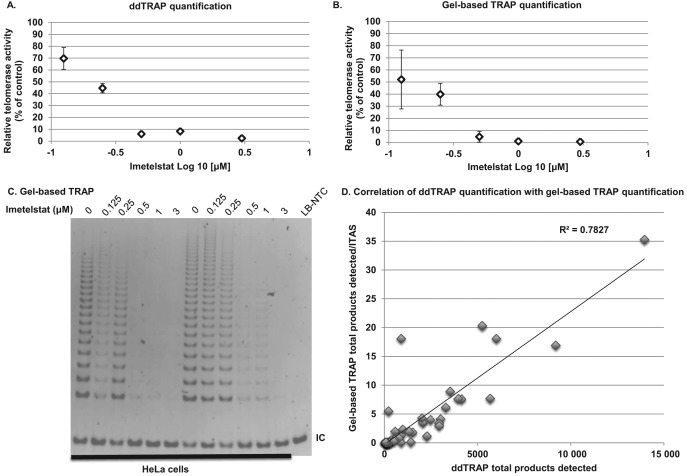
Comparison of ddTRAP to gel based TRAP: determination of Imetelstat IC50 in HeLa cells. ddTRAP quantification is less variable than gel based TRAP and allowed accurate determination of the IC50 of Imetelstat in HeLa (0.2 μM). HeLa cells were incubated with Imetelstat (0, 0.125, 0.25, 0.5, 1 and 3 μM) for 72 h. Cells were pelleted and triplicate extracts prepared from three separate tissue culture experiments (nine total extracts and extensions per dose). (**A**) ddTRAP quantification with 50 cell equivalents added to the PCR. (**B**) Gel quantification (representative gel image of two experiments in Figure 5C). (**C**) Gel based TRAP was performed with 125 cell equivalents. Data are expressed as relative telomerase activity compared to control (untreated HeLa) and standard error of the mean. (**D**) Correlation analysis of gel-based TRAP to ddTRAP. The *P* < 0.0001 positive relationship indicates that the methods are measuring the same phenomenon. ITAS = Internal competitive telomerase activity substrate.

We compared the gel based TRAP assay to ddTRAP in an experiment to determine the IC50 concentration of the telomerase RNA template antagonist, Imetelstat, in HeLa cells. The 2-fold variability of the gel based TRAP assay made it difficult to determine what dose of Imetelstat (24,25) was actually producing 50% inhibition (25), thus making accurate mechanistic studies of telomerase difficult. Both ddTRAP and gel-based TRAP show incremental decreases in telomerase activity with increasing dose of Imetelstat, indicating that the assays are detecting the same phenomenon (Figure 6). The correlation of ddTRAP to gel-based TRAP quantification indicated a strong relationship between the two measures (Figure 6D; *R*^2^ = 0.78, *P* < 0.0001). ddTRAP generated data with small variability (9.3 and 3.9% at 0.125 and 0.25 μM points, respectively) and allowed for accurate determination of the IC50 dose of Imetelstat in HeLa cells (ddTRAP determined Imetelstat IC50 dose = 0.2 μM; Figure 6A). However, using the gel-based TRAP quantification (quantification of gel images Figure 6B; representative gel image Figure 6C), the variability of the measures at the critical points (between 0.125 and 0.25 μM) to determine IC50 values was large enough (24.3 and 9.0%) to prevent accurate determination of the IC50 dose of Imetelstat (Figure 6B). These data highlight that traditional TRAP readily detects telomerase activity but is nearly impossible to use for quantitation of 20–30% differences due to large variability in gel-based quantification. Thus, ddTRAP is far superior in quantification of small but potentially biologically important differences in telomerase activity such as differences expected in loss of function experiments or in testing telomerase inhibition or activation strategies.

### Detection and quantitation of mouse telomerase activity with ddTRAP

**Figure 7.**
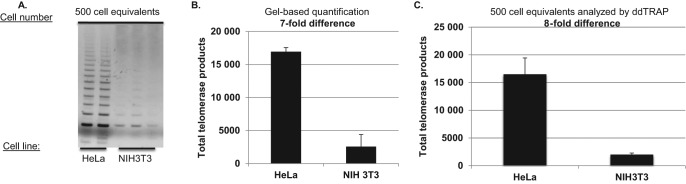
ddTRAP detection of mouse telomerase. Extracts from one million HeLa and one million NIH3T3 mouse fibroblasts were prepared. Following lysis (40 μl), 1-μl of the lysate (25 000 cell/μl) was added to a 50 μl extension reaction (extension product concentration = 500 cells/μl). One-microliter of the extension reaction was used for either the gel based TRAP or the ddTRAP assay. (**A**) Gel analysis (10% PAGE) of HeLa compared to NIH3T3 cells. Gel was stained with Gel red, a double stranded DNA binding dye. (**B**) ddTRAP total products generated (molecules per microliter output multiplied by 20 μl). Quantification showed an eight-fold difference in extension products Eqs. = equivalents. (**C**) Quantification of scanned images from A (ImageJ, http://imagej.nih.gov/ij/, (22)) showing a seven-fold difference which is similar to ddTRAP quantification.

There are sequence differences in the telomerase RNA component between human and mouse TERC (26). We examined whether ddTRAP, similar to gel based TRAP, was able to detect mouse telomerase activity using immortal NIH3T3 mouse fibroblasts (Figure 7). The magnitude of the difference of telomerase activity between TRAP and ddTRAP was similar in HeLa compared to NIH3T3 cells (Figure 7). Optimization of telomerase substrates that are more compatible with the mouse telomerase RNA component will be needed for determination of absolute molecule counts of mouse telomerase activity. The present data does not address how efficiently mouse telomerase works on the TS primer, but ddTRAP offers a suitable quantitative surrogate to traditional gel-based methods.

### Detection of telomerase activity in human lymphocytes

**Figure 8.**
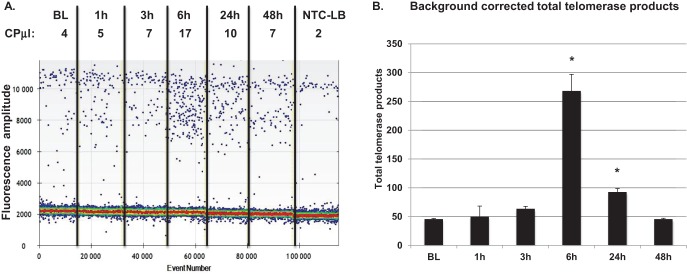
ddTRAP detects mitogen-stimulated telomerase in human PBMCs. Fifty cell equivalents were assayed using ddTRAP. (**A**) Raw ddPCR output. Telomerase activity was detected above background at 6 h of PHA stimulation that persisted until 24 h and returned to baseline levels at 48 h of stimulation. (**B**) Calculated total telomerase products generated during the extension reaction (ddPCR output in copies per microliter multiplied by 20). Data are background corrected. CPμl = copies of target per microliter, output from the droplet reader software. *Significantly different from control *P* < 0.05 by Student's Paired *t*-test.

Telomerase enzyme activity is limited to small subset of adult cell types such as transient-amplifying germ cells, proliferating adult stem cells and activated immune cells (20). Technical difficulties and limited samples hinder accurate quantitation of low levels of telomerase activity, such as telomerase activity in activated (i.e. via mitogen stimulation) human adult peripheral blood mononuclear cells (PBMCs). PBMCs from a healthy, physically active 29-year-old volunteer were stimulated with mitogen, which activates T-cells (20). Mitogen stimulation resulted in a peak in telomerase activity at 6 h post stimulation that persisted until 24 h and returned to baseline at 48 h post mitogen treatment (Figure 8). One needs upwards of 10 000 cell equivalents to perform these experiments with gel based TRAP assay (20). In contrast, the data in Figure 8 was generated from only 50 cell equivalents. These data show that ddTRAP is far superior compared to the gel based TRAP for quantitation of telomerase activity from samples with limited cell numbers.

## DISCUSSION

Our results demonstrate that a novel form of emulsion PCR, ddPCR, can quantify telomerase enzyme activity on a single-cell basis. This method allows precise quantification of telomerase enzyme activity from a variety of cell types, both tissue culture cell lines and primary adult human cells. The coefficient of variation is very small so that accurate and reproducible observations are possible. The ddTRAP assay permits quantitative evaluation of telomerase modulating interventions. While telomerase is one example, the methods described here provide a ‘blueprint’ for the adaptation of this powerful new technology (ddPCR) for studies of a wide variety of enzyme activity assays based on PCR.

Most techniques used to quantify telomerase enzyme activity provide relative values requiring internal standards and reference samples, methods of absolute determination of telomerase activity are lacking. The measurement of telomerase enzyme activity by the primer-extension assay is considered to be the gold standard for determination of telomerase activity (27,28). The primer-extension assay directly measures the addition of telomere repeats to a telomerase substrate without PCR and detects extension products via radiolabeling on a polyacrylamide gel (29). This method is powerful for measuring the processivity of telomerase, but the need for partial purification greatly reduces its ability to accurately quantify telomerase activity. Purification methods such as antibody or telomerase nucleotide substrate pull-downs require large amounts of sample material (>2 million cells), time and technical expertise (29). Using this method to measure telomerase activity in clinical biopsies is typically not feasible.

Other researchers have attempted to eliminate the PCR step or alter the detection method for the TRAP assay (30–39). Recently, gold plated nanoparticles with a bound telomerase substrate were used in combination with PCR to detect telomerase (38). Zheng *et al.* (39) also used a modified version of TRAP and detected telomerase activity in as few as 10 HeLa cells. Tian and Weizmann developed a rapid isothermal telomerase detection system, exponential isothermal amplification of telomere repeat (EXIALTR) that abandoned the thermocycling protocol, achieved single cell resolution and could be completed in about 25 min (33), however, EXIALTR is only relatively quantitative in comparison to a standard curve. The ddTRAP assay is simple to set-up and is far more quantitative compared to its gel-based counterpart. The adaptation of the TRAP assay to the ddPCR platform is a significant advance in our ability to quantitatively assay telomerase activity, even at the single-cell level, compared to other methods currently available.

The development of ddPCR and adaptation of the TRAP assay allows for accurate, standard free quantification of telomerase activity, using a non-radioactive method with sensitivity comparable to radiolabeling detection methods (13,16). ddTRAP relieves some of the concerns associated with traditional gel-based TRAP assays. Since each PCR reaction is occurring in a separate droplet, this eliminates PCR size bias and reduces the likelihood of off target-primed molecules from amplifying and interfering with on-target template priming and quantification. Further, the ddTRAP assay is linear without the addition of an internal competitive template, simplifying set-up and quantification of telomerase-extended products. Finally, the simple set-up and data output of the ddTRAP assay makes it more amenable applications such as moderate throughput screening of genes or chemicals associated with telomerase activity or discovery of novel telomerase manipulating targets. The ddTRAP assay accurately quantitates small changes (either increases or decreases) in telomerase that may be important for the development or tracking of telomerase modulating interventions for both regenerative medicine (increasing telomerase) and cancer therapies (decreasing telomerase).

ddTRAP also can use very small cell numbers from clinical samples. ddTRAP is able to reproducibly quantitate as little as a 15–20% change, which is a great improvement compared to the ∼2-fold sensitivity of the gel based TRAP assay. The adaptation of the TRAP assay to the droplet digital platform now provides a means to more completely study the manipulation of telomerase enzyme activity. The adaptation of the TRAP assay to the digital format is not meant to replace the gel-based TRAP assay. The gel-based TRAP assay is still sufficient for determination of absence or presence of telomerase activity and for qualitative comparisons between samples. Situations such as differentiation of stem cells to more mature progeny where a down regulation of telomerase is expected, gel-based TRAP would suffice (40). However, when mechanistic studies of telomerase activity are necessary, such as manipulation of alternative splicing or transcription of TERT, where a 20% difference in telomerase activity may be biologically important, ddTRAP will be far superior compared to gel-based TRAP at quantification of these types of small differences. Further, ddTRAP will be far superior in determination of IC50 values for telomerase activity inhibitors compared to gel-based TRAP due to the variability in quantification associated with such methods, as shown in Figure 6. Further, the fact that ddTRAP accurately counts the absolute number of telomerase-extended DNA molecules provides direct information concerning how inhibition or activation strategies could be mechanistically altering telomerase activity and ultimately telomere length homeostasis. ddTRAP can also be very useful in biochemical studies of telomerase activity, especially in determining kinetics and other important enzyme parameters that have proved challenging to quantify accurately for telomerase with other assays. Other uses of ddTRAP could be to quantitatively track telomerase activity through various purification procedures to more carefully determine active protein yields, where a 20% difference not detected by a gel-based method would become clearly apparent using ddTRAP.

Another important biological phenomenon that ddTRAP can analyze is the heterogeneity of telomerase regulation in cancer cell populations. Since the ddTRAP assay can detect telomerase activity in a single cell it can determine the clonal variation within tissue culture populations. The ddTRAP assay detected telomerase activity in 73% of individual HeLa cells. This demonstrates that different cells within the population had different telomerase activity. When Reddel's group expanded small HeLa cell clones, one third of them lacked telomerase activity (41). This is very close to the one quarter of single HeLa cells that lacked telomerase activity as measured by ddTRAP. The ability to acquire this information will be extremely useful in exploring tumor cell heterogeneity and the response to telomerase modifying therapies.

In summary, the ddTRAP method allows small numbers of primary normal human adult cells (even single cells) to be assayed quantitatively for telomerase activity. This technique lowers the threshold of detection so that telomerase modulating therapies and interventions (i.e. lifestyle interventions including dietary and/or exercise training) could reliably be assessed with ddTRAP.

## SUPPLEMENTARY DATA

Supplementary Data are available at NAR Online.

SUPPLEMENTARY DATA
