# Supplementary material for: Quantitative telomerase enzyme activity determination using droplet digital PCR with single cell resolution
Source: Nucleic Acids Res. 2014 May 26;42(13):e104. doi: 10.1093/nar/gku439 (PMC4117742; doi:10.1093/nar/gku439)
Supplement: SUPPLEMENTARY DATA [file supp_42_13_e104__index.html]

SUPPLEMENTARY DATA 

# Quantitative telomerase enzyme activity determination using droplet digital PCR with single cell resolution

## SUPPLEMENTARY DATA

**Files in this Data Supplement:**

- Supplemental Figures
