# Supplementary material for: Quantitative telomerase enzyme activity determination using droplet digital PCR with single cell resolution
Source: Nucleic Acids Res. 2014 May 26;42(13):e104. doi: 10.1093/nar/gku439 (PMC4117742; doi:10.1093/nar/gku439)

Supplemental figures

- **Supplemental Figure 1. PAGE analysis of ddTRAP products.**

HeLa cell extension products were made starting with 1250 cells (diluted from an initial 50,000 cell lysate) in a 50μL reaction giving a final concentration of 25 cells per μL. Serial 2-fold dilutions produced 12.5, 6.25, 3.125, 1.78, and 0.78 cells per microliter. Following thermocycling droplets were treated with phenol:chloroform to disrupt the droplets and extract the amplified DNA, which was then resolved on a 10% PAGE and visualized with Gel Red staining. The expected 6-bp ladder pattern was produced using ddPCR.

- **Supplemental Figure 2. Intra-and-inter-day ddTRAP variation in H1299 cells.**

A. Intra day variability was assessed by making 3 extension reaction dilution series and assaying telomerase activity on one plate (3 replicates per cell equivalent input). Data are plotted as total telomerase product (copies per microliter multiplied by 20) by cell equivalents. Error bars are standard deviation of the replicates on each plate. **B.** Inter day variability was assessed by running two assays on two separate days from the same three extension reactions described in A. Data are plotted as total telomerase product (copies per microliter multiplied by 20) by cell equivalents. Error bars are standard deviation of the replicates on each plate. **C.** Technical variation was assed by diluting the 100 cell equivalent sample 1:2 in a series of 6 samples. Telomerase activity was measured on two separate plates on the same day. Technical variation is plotted in copies per microliter (output from ddPCR software) by cell equivalents input on a log scale. Error bars are Poisson corrected 95% confidence intervals across the two plates.

- **Supplemental Figure 3. HeLa cell linearity up to 500 cell equivalents input.**

Triplicate extension reactions were made in a dilution series from 500 to 15.625 cell equivalents by a factor of 2 and ddPCR performed. These data indicate that ddTRAP is linear for inputs up to 500 cell equivalents in HeLa cells. Lysates are from a HeLa clone specific to our laboratory and observed to have intermediate telomerase activity compared to other HeLa lines (See Figures 4 and 6 for HeLa population telomerase activity data).

- **Supplemental Figure 4. Gel-based TRAP images.**

Supplemental Figure 1. PAGE analysis of ddTRAP products

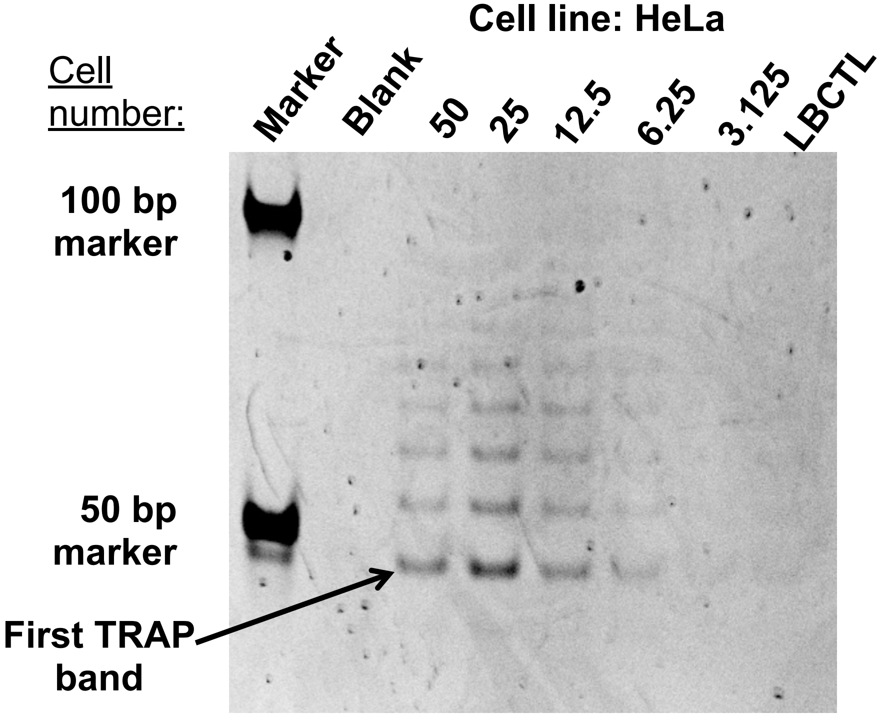

Supplemental Figure 1. PAGE analysis of ddTRAP products.
HeLa cell extension products were made starting with 1250 cells (diluted from an initial 50,000 cell lysate) in a 50μL reaction giving a final concentration of 25 cells per μL. Serial 2-fold dilutions produced 12.5, 6.25, 3.125, 1.78, and 0.78 cells per microliter. Following thermocycling droplets were treated with phenol:chloroform to disrupt the droplets and extract the amplified DNA, which was then resolved on a 10% PAGE and visualized with Gel Red staining. The expected 6-bp ladder pattern was produced using ddPCR.

Supplemental Figure 2. Reproducibility and linearity of ddTRAP in H1299 cells

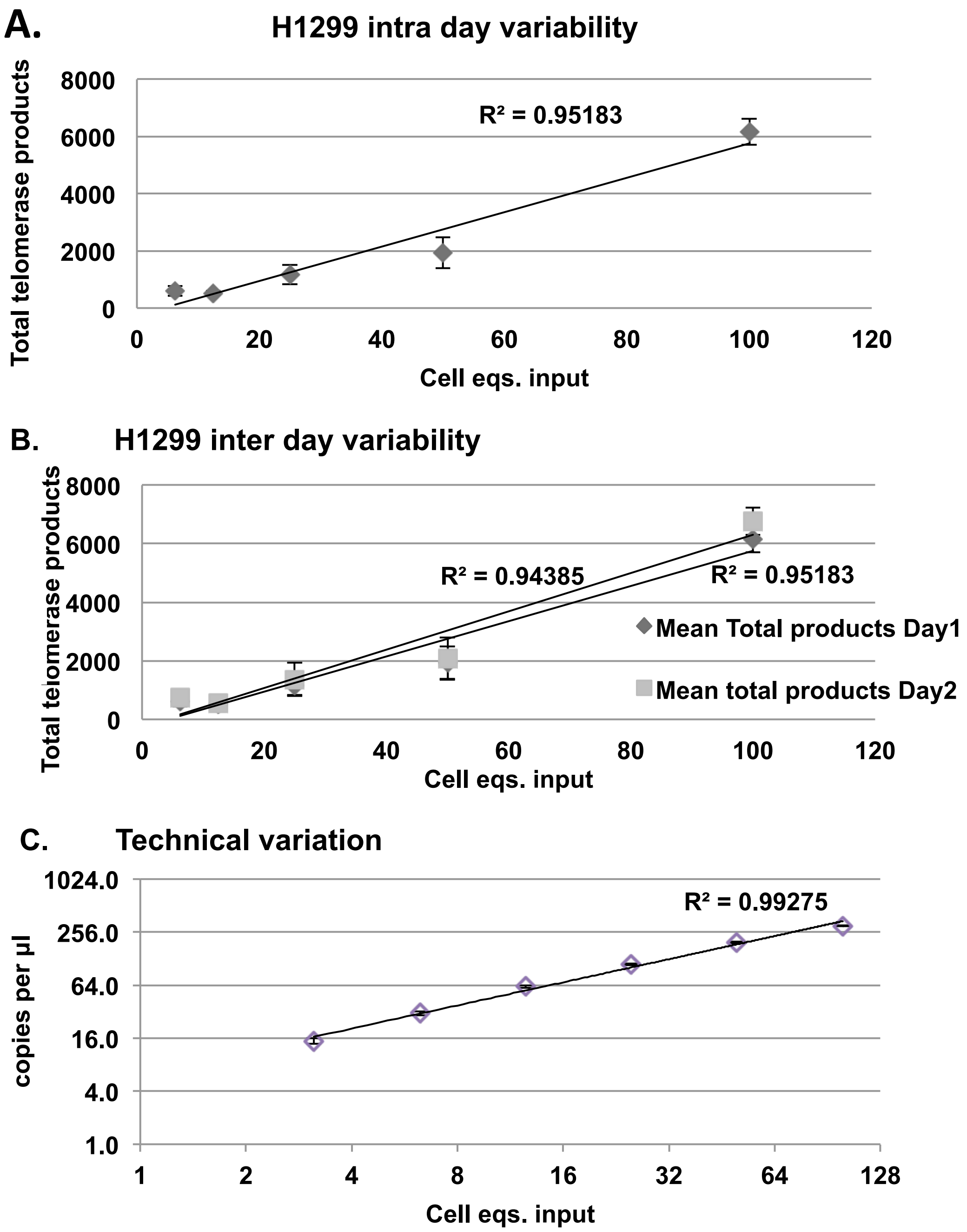

Supplemental Figure 2. Intra-and-inter-day ddTRAP variation in H1299 cells.
A. Intra day variability was assessed by making 3 extension reaction dilution series and assaying telomerase activity on one plate (3 replicates per cell equivalent input). Data are plotted as total telomerase product (copies per microliter multiplied by 20) by cell equivalents. Error bars are standard deviation of the replicates on each plate. **B.** Inter day variability was assessed by running two assays on two separate days from the same three extension reactions described in A. Data are plotted as total telomerase product (copies per microliter multiplied by 20) by cell equivalents. Error bars are standard deviation of the replicates on each plate. **C.** Technical variation was assed by diluting the 100 cell equivalent sample 1:2 in a series of 6 samples. Telomerase activity was measured on two separate plates on the same day. Technical variation is plotted in copies per microliter (output from ddPCR software) by cell equivalents input on a log scale. Error bars are Poisson corrected 95% confidence intervals across the two plates.

Supplemental Figure 3. Linearity of ddTRAP in HeLa cells up to 500 cell eqs. input

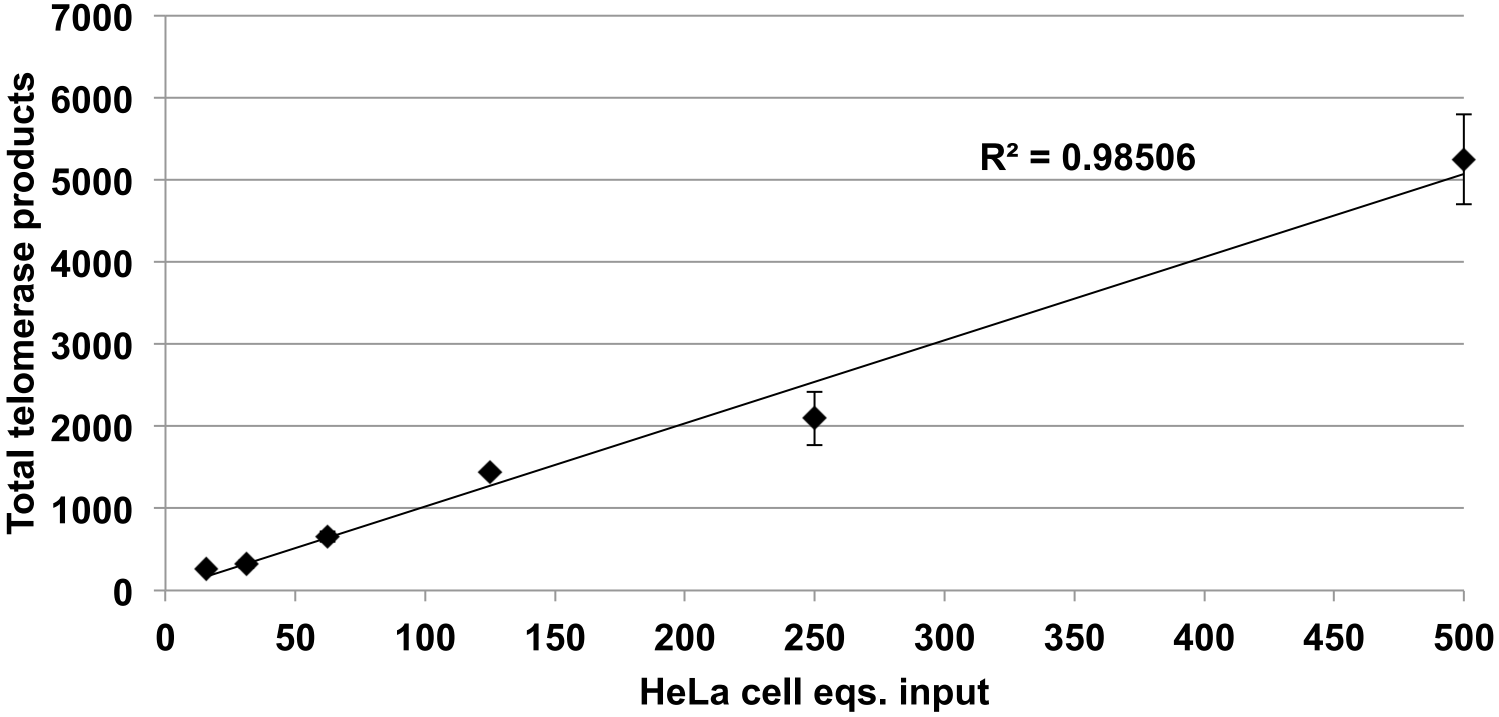

Supplemental Figure 3. HeLa cell linearity up to 500 cell equivalents input.
Triplicate extension reactions were made in a dilution series from 500 to 15.625 cell equivalents by a factor of 2 and ddPCR performed. These data indicate that ddTRAP is linear for inputs up to 500 cell equivalents in HeLa cells. Lysates are from a HeLa clone specific to our laboratory and observed to have intermediate telomerase activity compared to other HeLa lines (See Figures 4 and 6 for HeLa population telomerase activity data).

Supplemental Figure 4. Gel-based TRAP Images

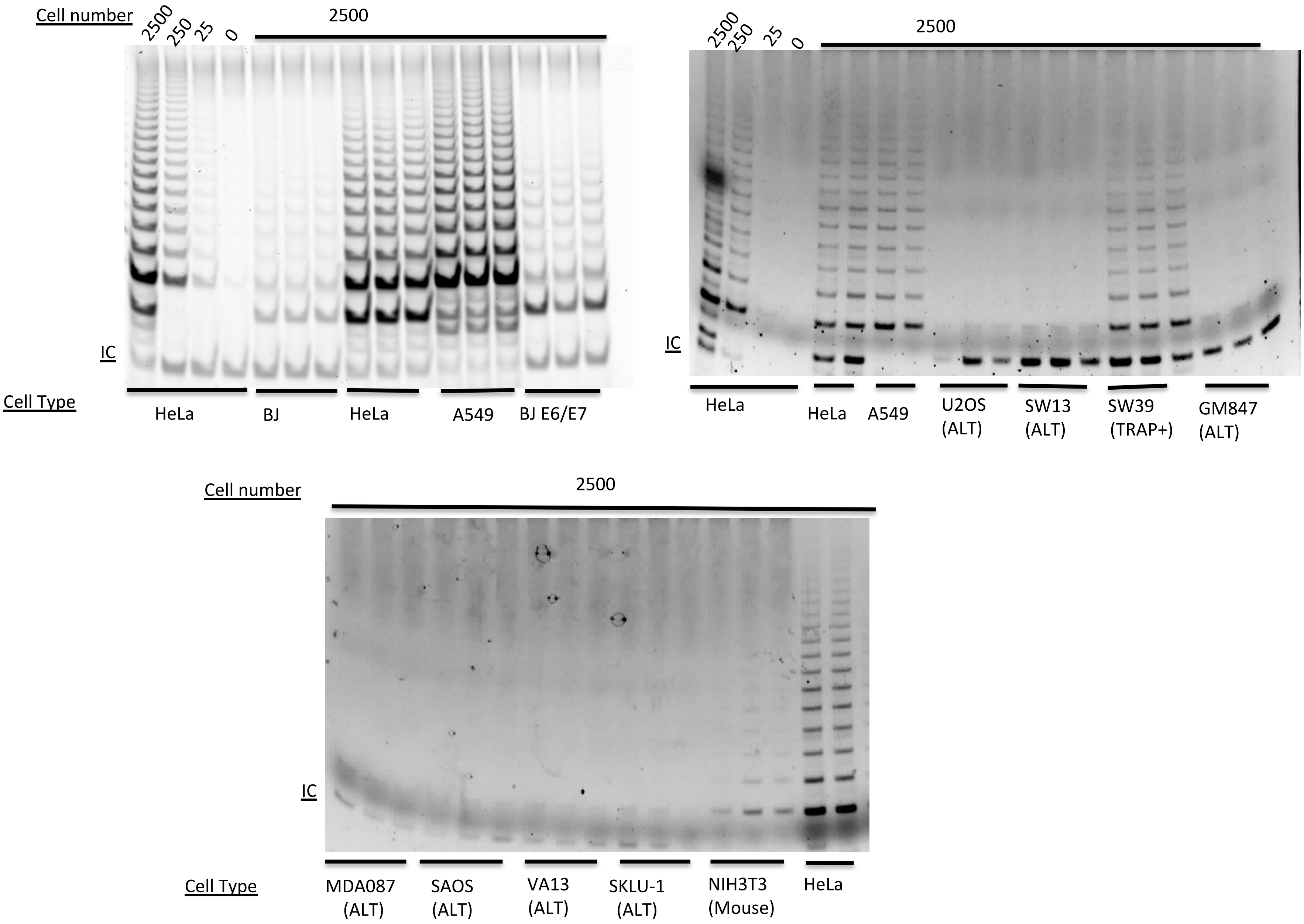

Supplement: SUPPLEMENTARY DATA [file supp_gku439_nar-02809-met-g-2013-File002.pdf]
